# Oleoylethanolamide treatment affects gut microbiota composition and the expression of intestinal cytokines in Peyer’s patches of mice

**DOI:** 10.1038/s41598-018-32925-x

**Published:** 2018-10-05

**Authors:** Monica Di Paola, Elena Bonechi, Gustavo Provensi, Alessia Costa, Gerard Clarke, Clara Ballerini, Carlotta De Filippo, M. Beatrice Passani

**Affiliations:** 1 Dipartimento di Biologia, Università di Firenze, Firenze, Italy; 2 Dipartimento di Neuroscienze, Psicologia, Area del Farmaco e Salute del Bambino, Universitá di Firenze, Firenze, Italy; 3 Dipartimento di Scienze della Salute, Università di Firenze, Firenze, Italy; 4 Department of Psychiatry and Neurobehavioural Science, and APC Microbiome Ireland, University College Cork, Cork, Ireland; 5 Dipartimento di Medicina Sperimentale e Clinica, Università di Firenze, Firenze, Italy; 6 Instituto di Biologia e Biotecnologie Agrarie (IBBA), Consiglio Nazionale delle Ricerce (CNR), Pisa, Italy

## Abstract

The lipid sensor oleoylethanolamide (OEA), an endogenous high-affinity agonist of peroxisome proliferator-activated receptor-α (PPAR-α) secreted in the proximal intestine, is endowed with several distinctive homeostatic properties, such as control of appetite, anti-inflammatory activity, stimulation of lipolysis and fatty acid oxidation. When administered exogenously, OEA has beneficial effects in several cognitive paradigms; therefore, in all respects, OEA can be considered a hormone of the gut-brain axis. Here we report an unexplored modulatory effect of OEA on the intestinal microbiota and on immune response. Our study shows for the first time that sub-chronic OEA administration to mice fed a normal chow pellet diet, changes the faecal microbiota profile, shifting the Firmicutes:Bacteroidetes ratio in favour of Bacteroidetes (in particular *Bacteroides* genus) and decreasing Firmicutes (*Lactobacillus*), and reduces intestinal cytokines expression by immune cells isolated from Peyer’s patches. Our results suggest that sub-chronic OEA treatment modulates gut microbiota composition towards a “lean-like phenotype”, and polarises gut-specific immune responses mimicking the effect of a diet low in fat and high in polysaccharides content.

## Introduction

Gut microorganisms are indispensable for the regulation of the host metabolism by protecting the intestine against exogenous pathogens and potentially harmful resident microorganisms. Several mechanisms converge to establish a healthy status of the host, which include a constant dialogue between microbiota and intestinal immune system. The intestinal microbial community plays a pivotal role in the development of the innate immune system and is essential in shaping adaptive immunity^1^. In turn, intestinal immune responses regulate the composition of the microbiota^2,3^. The intestinal immune system has particular features to control the proliferation and composition of intestinal microbes. Peyer’s patches are gut-associated lymphoid follicles that, by their ability to transport luminal antigens and bacteria, can be considered as the immune sensors of the intestine. Peyer’s patches functions, such as induction of immune tolerance or defence against pathogens, result from the complex interplay between immune cells located in the lymphoid follicles and the follicle-associated epithelium. Hence, Peyer’s patches sampling of the lumen is crucial for protective mucosal immune responses^4^. Dysbiosis disrupts gut homeostasis and increases the risk of inflammatory responses and it is now broadly accepted that changes in the microbiota are associated with not only intestinal^5^, but also immunological and metabolic diseases^6^. In addition, changes in the microbiota may affect the central nervous system through the gut-brain axis and may contribute to signs and symptoms of neurological diseases^7–10^. Diet has a fundamental role in shaping the gut microbiota profile; for instance, an animal-fat based diet leads to fall in bacterial diversity and shifts the proportions of the two most abundant phyla of gut microbiota, Firmicutes and Bacteroidetes^11^. On the contrary, a fiber-rich diet promotes bacterial diversity that affords an anti-inflammatory outcome, as it ameliorates pathological profiles as for instance in type-2 diabetes^12^. It has been proposed that diet-induced obesity is associated with dysbiosis that creates a permissive gut inflammatory environment^13^. Although the literature reports some discrepancies regarding how the inflammatory state in the intestine relates to obesity and metabolic diseases, some studies have shown that obesity is associated with an increased intestinal inflammation and elevated levels of intestinal T_H_1 and T_H_17 lymphocytes at the relative expense of T_H_2 and anti-inflammatory T regulatory (Treg) lymphocytes. For instance, Treg levels decrease following prolonged high fat diet in mice^14,15^.

A newly discovered player in the homeostasis of intestinal functions is the endocannabinoid system^16^. In this regard, oleoylethanolamide (OEA) a potent agonist of PPAR-α, has a prominent role in gut physiology^17^. OEA is synthesized in the gastro-intestinal tract and was first described as a fat sensor that mediates satiety^18,19^. In addition, the levels of OEA activation may change in response to inflammation^20^, diet^21,22^ or food intake^18,23,24^. A vast literature suggests that endogenous acylethanolamides, signalling through PPAR-α exert a tonic inhibitory control on the induction of nociception and inflammation therefore helping to maintain host-defence homeostasis by preventing inappropriate reactions^25,26^. OEA is mobilized in the proximal intestine after a meal^27^, but it is currently unclear whether it also affects intestinal homeostasis by changing the profile of the microbiota and intestinal lymphocytes activity. To answer these questions, we examined the consequences of sub-chronic OEA administration on the faecal microbiota profile and intestinal cytokines expression by immune cells of the mouse Peyer’s Patches.

## Results

### OEA treatment decreases body weight and food consumption

**Figure 1:**
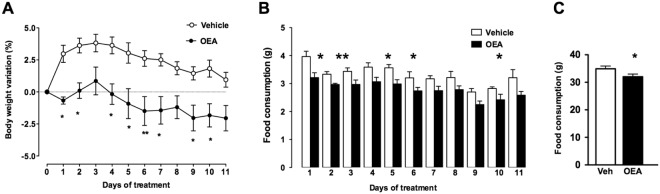
Effect of sub-chronic OEA administration on body weight and food consumption. (**A**) Time course of the effects of vehicle (VEH) or OEA (10 mg/kg, i.p.) on body weight change in mice. Two-way ANOVA with repeated measures and Bonferroni’s post hoc test; significantly different from vehicle treated mice, *P < 0.05; **P < 0.01; (**B**) Time course of the daily and (**C**) Cumulative food intake. Student’s T test, *P < 0.05; **P < 0.01; n = 9–13 per group.

As shown in Fig. 1A mice treated with OEA (10 mg/kg i.p.) gained significantly less weight than control mice treated with vehicle (2 way Anova repeated measures and Bonferroni post hoc test; F_days×treatment 11,220_ = 2.217, P < 0.05 F_days 11,220_ = 6.126, P < 0.001; F_treatment 1,220_ = 11.59, P < 0.01; n = 9–13), although this difference was lost on the last day of treatment. The modest weight loss was associated with a moderate but significant reduction of food intake (Fig. 1B,C. Unpaired Student’s t test; *P < 0.05; **P < 0.01). Hence, mice did not develop tolerance to the anorexic effect of OEA, as previously reported in rats^19^.

### Effect of OEA sub-chronic treatment on gut microbiota profiles in mice

In order to evaluate the effect of OEA sub-chronic treatment on gut microbiota, we analysed faecal microbial profiles in a subgroup of mice before treatment at steady state (T0; n = 14) and after treatment with OEA 10 mg/kg, i.p. for 11 days (OEA_T11; n = 7). We then compared the microbiota composition of OEA-treated mice with that of vehicle-treated mice (VEH_T11 group; n = 7), used as controls.

**Figure 2:**
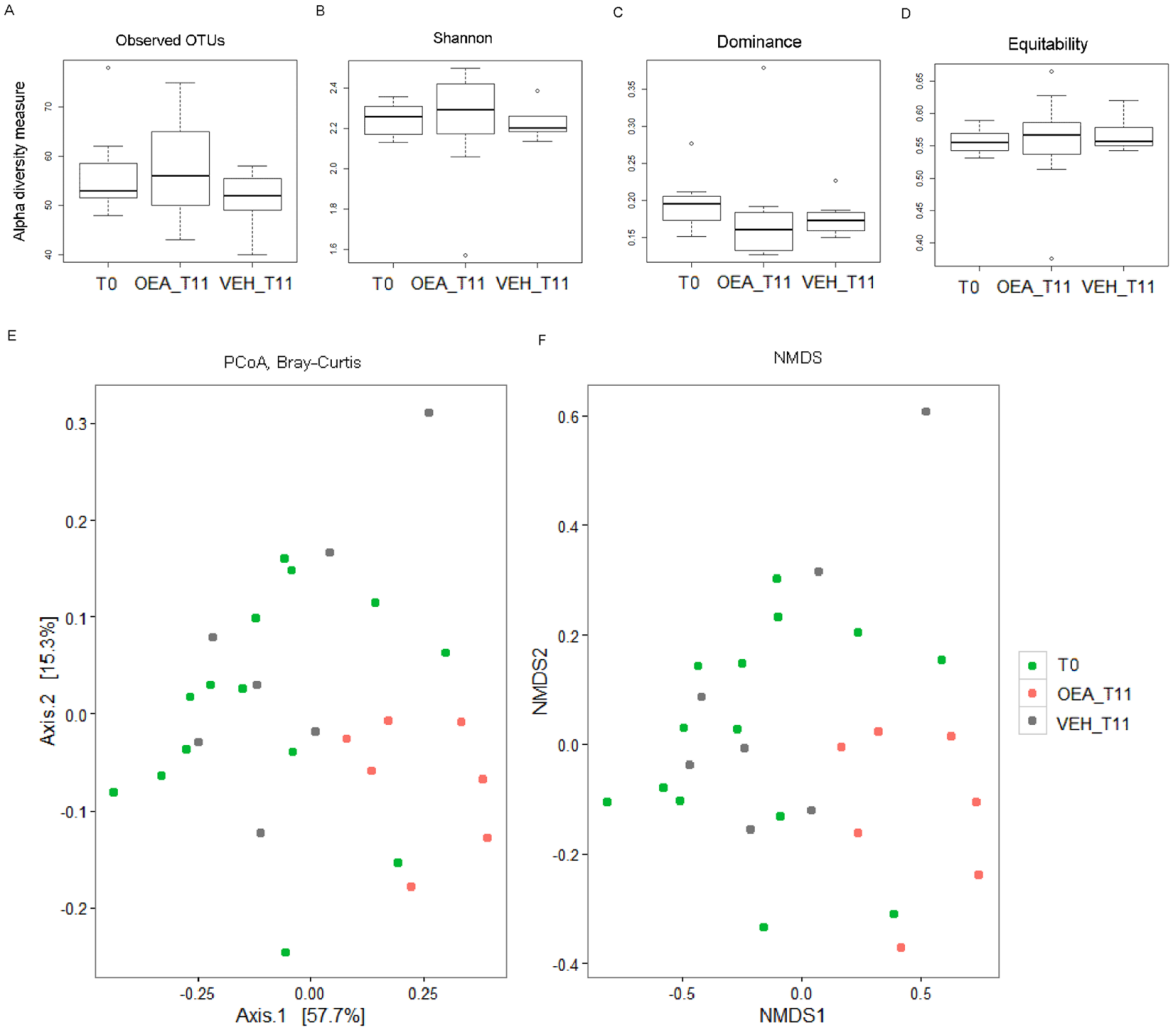
Alpha and Beta diversity. (**A**–**D**) Alpha diversity measures. Box plots of (**A**) observed OTUs, (**B**) Shannon index, (**C**) Dominance, and (**E**) Equitability calculated for mice groups at T0 = before the treatment, after 11 days of OEA treatment (OEA_T11) and after 11 days of vehicle treatment (Vehicle_ T11). Pairwise comparisons by using the Wilcoxon rank sum test were not significant. (E-F) Beta diversity measure. (**E**) PCoA, and (**F**) NMDS based on Bray Curtis dissimilarities. Samples at T0, samples of OEA_T11 and Vehicle_T11 are indicated with different colours. P = 0.035 PERMANOVA using the Adonis function with 999 permutations. For NMDS, stress value=0.09 indicated a good representation.

Alpha diversity was estimated by observed OTUs (a measure of microbial richness), Shannon entropy, a measure of entropy accounting for both abundance and evenness of bacterial species, Dominance, an index indicating whether one taxon dominates the community completely, and Equitability, a measure of the evenness with which bacterial species are divided among the taxa (see Methods). Although the differences were not statistical significant, the observed OTUs showed a trend of increased species richness in OEA-treated mice (T11) compared to T0, and a trend of higher microbial diversity (as indicated by Shannon index) after OEA treatment (T11) compared to T0 and vehicle-treated mice (Fig. 2A,B). We also observed that no taxon dominates bacterial community after OEA treatment, as observed by Dominance index, and that no differences in evenness of bacterial communities were found among T0, OEA treatment and controls, as observed by Equitability index (Fig. 2C,D).

To evaluate differences in faecal microbiota communities between groups, we analysed beta diversity by using Non-metric Multi-Dimensional Scaling (NMDS) and Principal Coordinates Analysis (PCoA) on Bray-Curtis distances. Both ordinations showed a clear separation of OEA-treated mice samples with respect to samples at T0 and that of vehicle-treated mice (Fig. 2E,F; P = 0.035 PERMANOVA), suggesting an effect of OEA on gut microbiota profiles.

**Figure 3:**
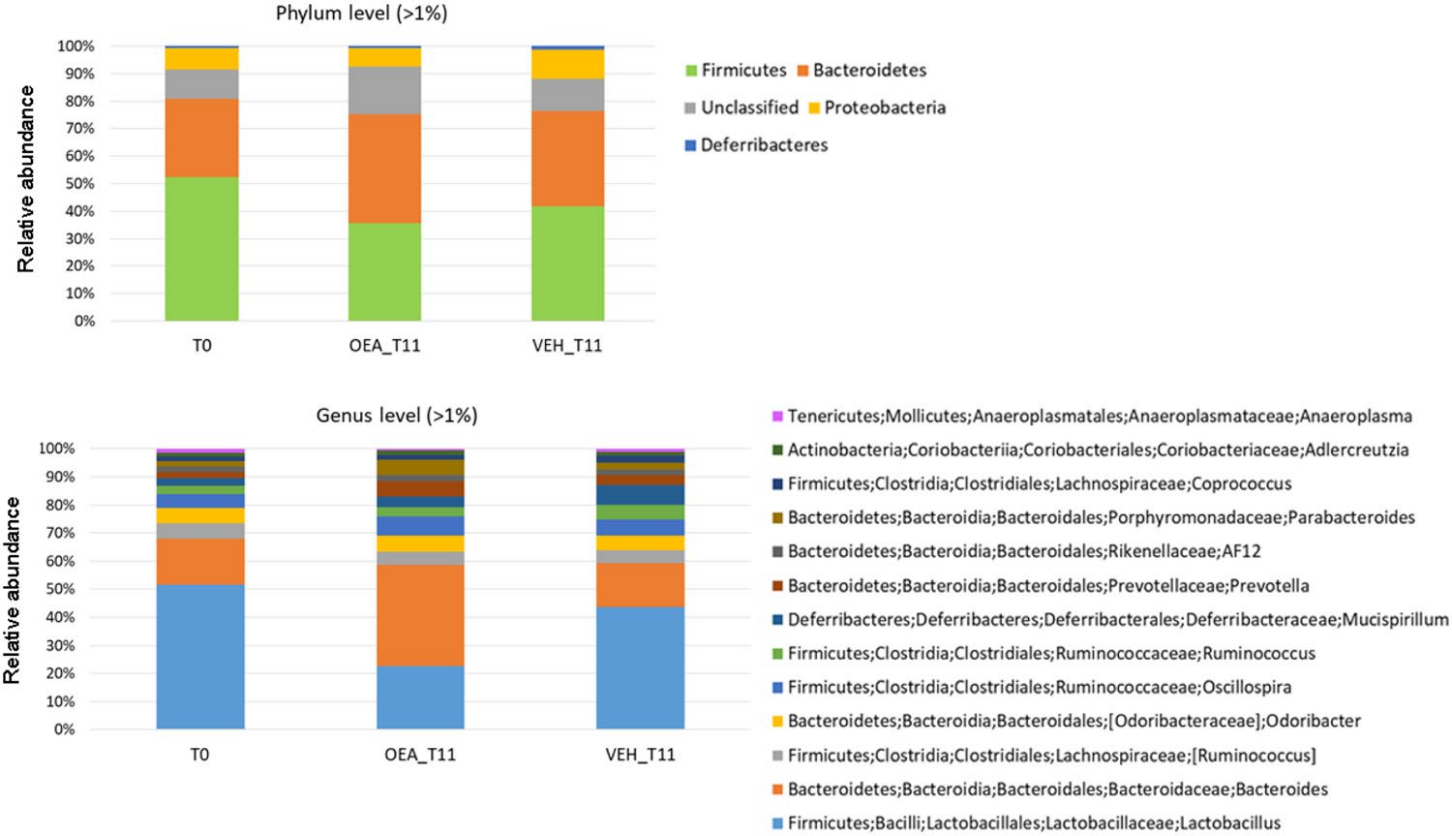
Barplot representation of microbial profiles. Relative abundances, on average, at the phylum and genus level of the microbiota in mice before the treatment (T0), after OEA and vehicle treatment at T11.

We then performed a meta-taxonomic analysis at different taxonomic levels. In Fig. 3, the most abundant bacterial phyla and genera (>1%) in mice at T0 and after OEA treatment and in controls at T11 are shown.

**Figure 4:**
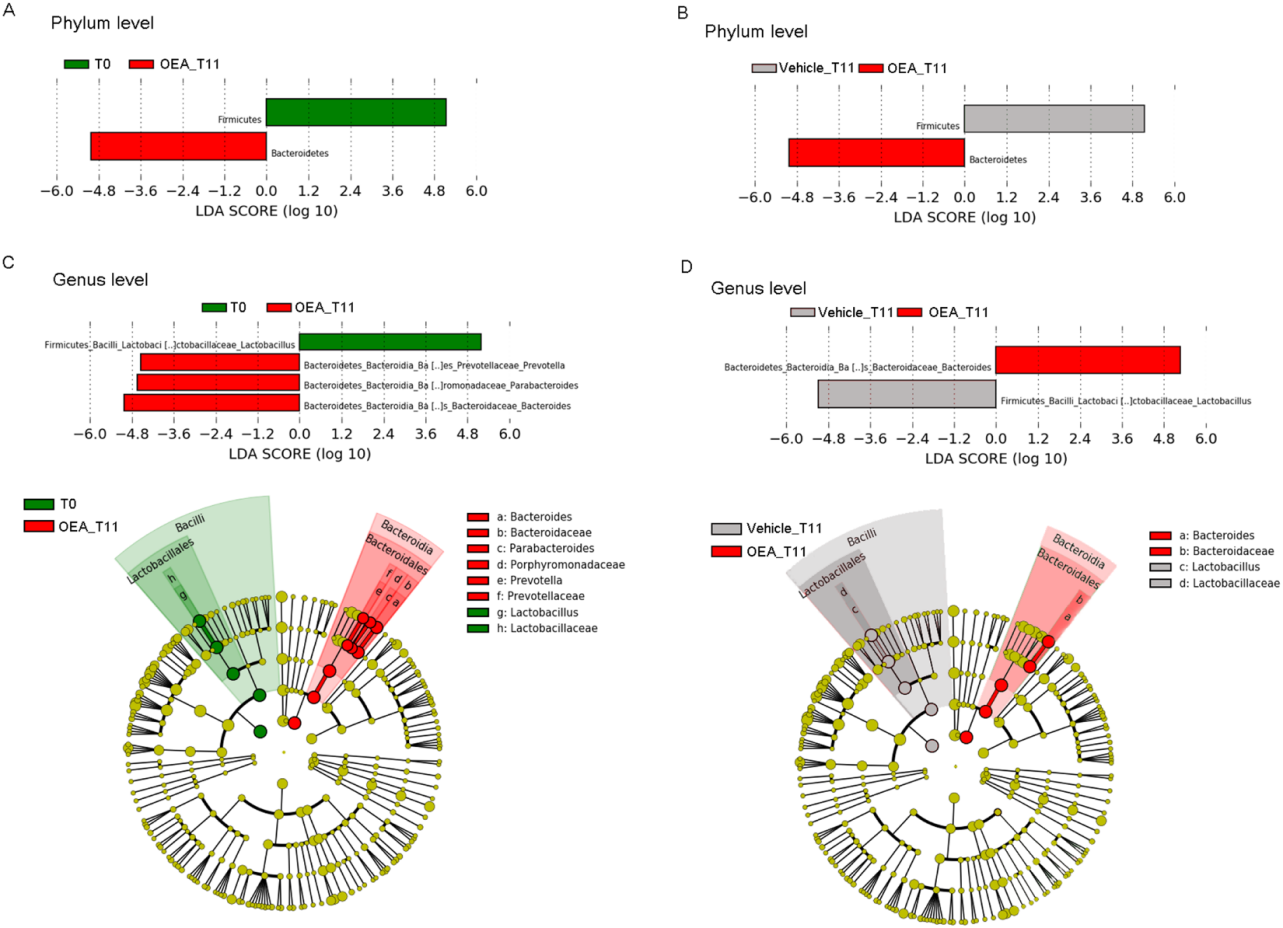
Metagenomic biomarker discovery by LEfSe analysis. Differential enriched bacterial taxa at the phylum level in gut microbiota of mice (A) before (T0) and after OEA treatment (T11) and (**B**) of mice treated for 11 days with OEA or vehicle- (Vehicle_T11). Differentially enriched bacterial taxa at the genus level in the gut microbiota of mice (C) before (T0) and after OEA treatment (T11) and (D) 11 days after OEA (T11) or vehicle treatment (Vehicle_T11). In the lower panels, cladograms show the most discriminative bacterial clades. Coloured regions/branches indicate differences in the bacterial population structure between the different groups. Statistically significant taxa enrichment among groups was obtained with Kruskal-Wallis test among classes (Alpha value = 0.05). The threshold for the logarithmic LDA score was 2.0.

Linear discriminant analysis Effect Size analysis (LEfSe) showed differentially enriched phyla at T0 and after OEA treatment (OEA_T11; Fig. 4A). Firmicutes were enriched at T0 and in vehicle-treated mice at T11 (relative abundance on average, 52% at T0 and 41% for vehicle treated mice vs 35% after OEA treatment; Fig. 4B). Bacteroidetes on the other hand, were enriched at T11 after OEA treatment when compared with both T0 (on average, 39% after OEA treatment vs 28% at T0) and with vehicle treated mice at T11 (34%).

At the genus level, we observed that, when compared to T0, OEA treatment enriched the microbiota of *Bacteroides* (on average 16% at T0 vs 34% at T11 of OEA-treated mice)*, Prevotella* (on average 2% at T0 vs 5% at T11 of OEA-treated mice), and *Parabacteroides* (on average 2% at T0 vs 5% at T11 OEA-treated mice; Fig. 4C). When compared to vehicle-treated mice, *Bacteroides* were enriched in OEA-treated mice (34% OEA treated-mice vs 15% control mice; Fig. 4D), whereas the observed different abundances of *Prevotella* and *Parabacteroides* between vehicle- and OEA-treated mice at T11 were not significant (Fig. 4D). Sequence alignments by BLASTn indicated that the majority of *Bacteroides* sequences in microbiota of OEA-treated mice were attributable with 97–98% of identity (see section “Methods”) to *B. acidifaciens* and to a lesser extent to *B. sartorii*.

Furthermore, LEfSe analysis showed that *Lactobacillus* was significantly reduced after OEA treatment, in comparison with either T0 or vehicle-treated mice at T11 (22% vs 49% vs 42%, respectively; Fig. 4C,D). Sequence alignments by BLASTn indicated that *Lactobacillus* sequences were attributable from 97% to 99% of identity to different species, especially *L. reuteri* and *L. gasseri*, and to a lesser extent to *L. murinus* and *L. johnsonii*.

### Prediction of functional metabolic profiles of gut microbiota in OEA-treated mice

**Figure 5:**
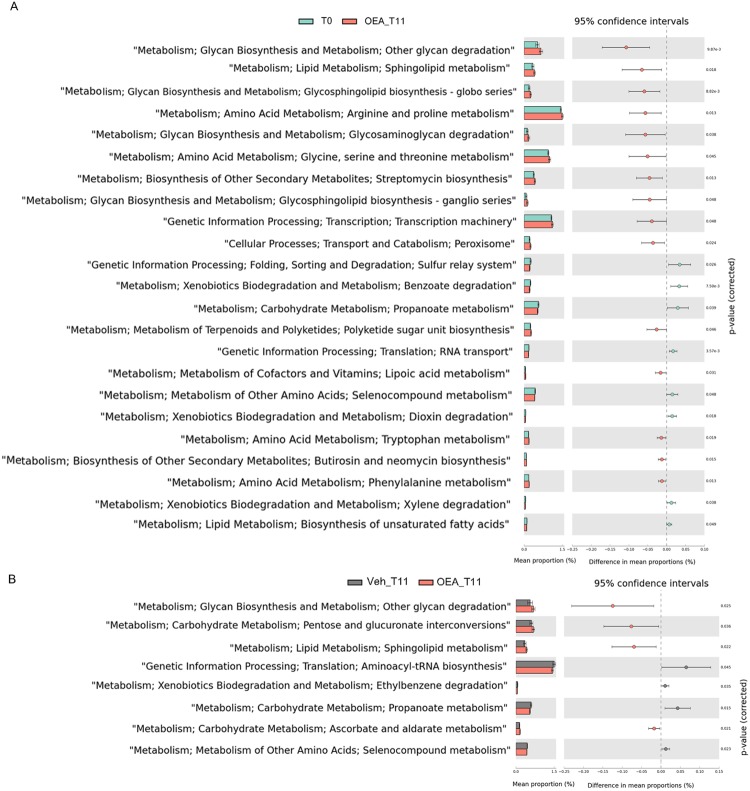
PICRUSt functional analyses. Extended error bar plots representing (**A**) Microbial pathways predicted to be differentially enriched in microbiomes of mice at T0 and after OEA treatment (OEA_T11). (**B**) Microbial pathways predicted to be differentially enriched in microbiomes of mice after OEA treatment (OEA_T11) versus controls (Veh_T11). Each extended error bar plot indicates the p-value along with the effect size and the associated difference in mean proportion and confidence interval for each predicted KEGG function. Each bar plot indicates the mean proportion of sequences assigned to the KEGG categories in each group. P-values by White’s nonparametric t-test, Storey FDR correction.

In order to predict how the observed differences in microbial profiles affected by OEA treatment, reflect differentially enriched functional pathways in mice, we applied PICRUSt (Phylogenetic Investigation of Communities by Reconstruction of Unobserved States). We observed functional classes (KEGG categories) differentially enriched in mice at T0 and after OEA treatment at T11 (Fig. 5A). In particular, among the metabolic functions significantly enriched in the microbiome of OEA-treated mice compared to T0, we found KEGG categories related to amino acids metabolism, (such as arginine and proline metabolism, glycine, serine and threonine metabolism, phenylalanine metabolism and tryptophan metabolism), together with functions related to glycan biosynthesis and metabolism (including functions related to other glycan degradation and glycosaminoglycan degradation). Other significant enriched functions were sphingolipid metabolism, glycosphingolipid metabolism and lipoic acid metabolism. Functions related to carbohydrate metabolism (such as butanoate metabolism and propanoate metabolism), and lipid metabolism (such as biosynthesis of unsaturated fatty acids), were reduced in OEA-treated mice compared to T0.

When vehicle and OEA treatment were compared at T11 (Fig. 5B), the metabolic functions enriched in the microbiome of OEA-treated mice regarded the metabolism of simple sugars and polysaccharides (such as pentose and glucoronate interconversions and other glycan degradation), and sphingolipid metabolism, whereas propanoate metabolism was reduced.

### OEA sub-chronic treatment modulates cytokines expression in intestinal Peyer’s patches

Peyer’s patches, together with small intestine epithelial cells, are thought to be the first station for the discrimination between pathogens and commensal bacteria. As OEA modified the microbiota profile, we also investigated Peyer’s patches response to the sub-chronic administration of OEA. In order to study cytokine production of Peyer’s patches isolated cells, we tested two mitogens that activate different immune cell populations. Isolated Peyer’s patches cells were incubated in either the lymphocyte mitogen phytohaemmagglutin (PHA), that mainly induces IL2, IFNγ and TNFα production by indirect T cell receptor cross linking; the other mitogen tested was lipopolysaccharide (LPS) that induces the production of critical proinflammatory cytokines necessary to activate immune responses by binding to TLR4 and triggers the NFkB pathway in innate immune cells (e.g. dendritic cells, macrophages), lymphocytes and intestinal epithelial cells (IECs, reviewed in^28^).

**Figure 6:**
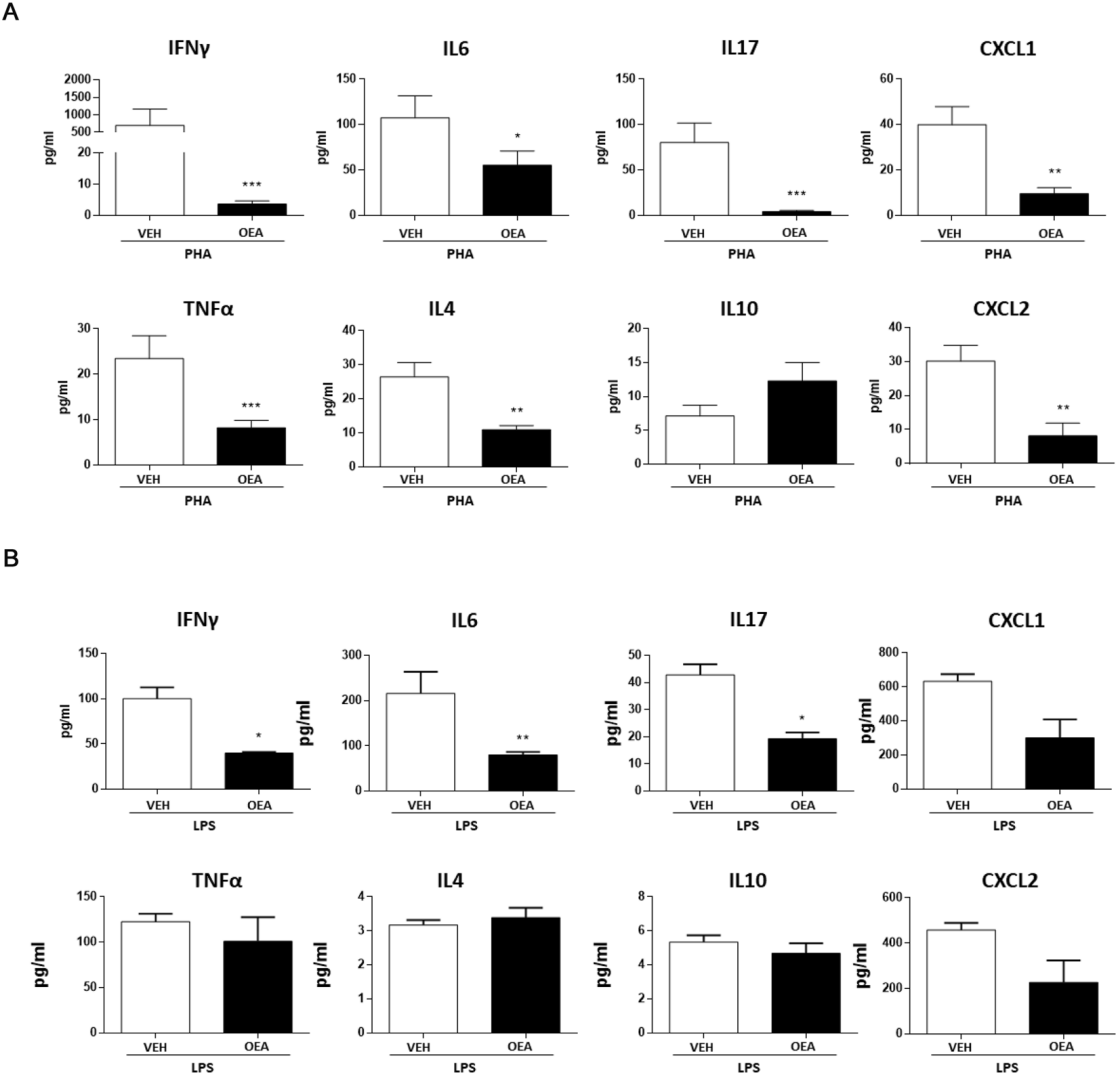
Changes of cytokines and chemokines secretion in intestinal Peyer’s Patches. Protein production was determined by Luminex technology on cells supernatant of OEA- (black column) and vehicle-treated mice (withe column). Cell were activated by either PHA (5 µg/ml) (**A**) or LPS (1 µg/ml) (**B**). Each histogram represents mean value ± SEM of protein concentration (pg/ml) of 12 samples for each group of PHA condition from 3 independent experiments and 4 samples for each group of LPS from 1 experiment. Statistical significance of the differences between VEH- and OEA-treated mice was analysed using Student’s t-test; *P < 0.05, **P < 0.01, ***P < 0.001.

As shown in Fig. 6A, a variety of cytokines and chemokines released by lymphocytes were differentially affected by OEA treatment. Cytokine regulation in response to OEA included a significant decrease of pro-inflammatory IFNγ, IL6, IL17, IL4, TNFα compared to vehicle treated mice (Unpaired Student’s t test, *p < 0.01, **p < 0.001, ***p < 0.0001; n = 5 animals X group). We observed a trend of IL10 increase that did not reach statistical significance. Furthermore, OEA decreased the release of the chemokines CXCL1 and CXCL2 that are required for recruitment of neutrophils during inflammatory responses following bacterial infections and injury^29^. In the presence of LPS, immune cells from Peyer’s patches of OEA-treated mice produced significantly less IFNγ, IL6, IL17, whereas no significant differences were observed for the other cytokines and for chemokines (Fig. 6B; unpaired Student’s T test, *P < 0.05, **P < 0.01, ***P < 0.001).

### Treatment with OEA modifies lymphocytes transcription factor expression

**Figure 7:**
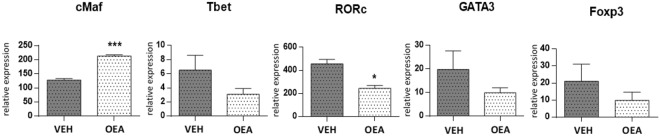
*Ex vivo* analysis of cells isolated from intestinal Peyer’s Patches. Relative expression of transcription factors by real time PCR. The graph shows mean values ± SD of 4 vehicle-treated (dark column) and 4 OEA-treated mice (light column) of two independent experiments. Expression of transcription factors is reported as ratio to ubiquitin. Student’s t-test, **p = 0.03; ***p < 0.001.

We also evaluated if the modulation of lymphocytic cytokines production was due to the activation of different subsets of T lymphocytes by analysing the relative expression of cMaf, Tbet, RORc GATA3 and FOXP3, transcription factors that affect the functional capabilities

and flexibility of CD4^+^ T cell subsets^30^. We found that OEA treatment significantly increased cMaf associated with T_H_2 (IL10 producing lymphocytes) and macrophages^6,31,32^ and decreased RORc (associated with T_H_17 lymphocytes) expression (Fig. 7; Unpaired Student’s t test, ***P < 0.001 and **P = 0.03), whereas the observed decrease of T_H_1 associated Tbet, GATA3 (T_H_2 lineage-specifying factor) and Foxp3 (Treg lineage-specifying factor) did not reach statistical significance. These results strongly suggest a trend towards T_H_2 polarization at the expenses of T_H_17, an observation also corroborated by the significant decrease of IL17, IL6 expression in the Peyer’s patches of OEA-treated mice.

## Discussion

This study shows for the first time that OEA treatment changes the microbiota composition of mice in a way that mimics the effect of a diet low in fat and high in polysaccharides content, and is also able to polarise gut-specific immune responses. We also confirmed that OEA treatment is associated with the reduction of body weight and, in agreement with previously reported results^19,23^, we observed that the change in body weight was associated with a small reduction of food consumption. In our hands, the overall effects of OEA are in line with those reported by other authors in rats and mice^19,33,34^. When comparing our results to those of other authors though, OEA treatment did not have the same effect on body weight change at all time points. Different experimental settings such as difference in the day time at which OEA was administered, animal strains, single-caging may be responsible of the discrepancies.

Compared with steady state (T0) and control treatment (vehicle at T11), sub-chronic OEA treatment modified microbial profiles in faeces of mice, as observed by analysis of biodiversity. This diversity is reflected in the populations of the gut microbiota, as OEA shifted the Firmicutes:Bacteroidetes ratio in favour of Bacteroidetes, in particular the genus *Bacteroides*, and reduced Firmicutes, in particular the genus *Lactobacillus*. This Firmicutes:Bacteroidetes trend was observed for the first time in *ob/ob* versus lean mice by Ley and co-workers^35^ and subsequently in human obese subjects^36^. These effects of OEA on the microbiota profile are very much like those afforded by a fiber-rich diet that promotes the survival of saccharolytic bacteria, such as *Bacteroides*, able to use dietary-derived glycans as energy sources, thanks to the high number of glycoside hydrolases and polysaccharide lyases (reviewed in^11,37^).

In agreement with the body weight reduction induced by OEA, a previous study demonstrated that *Bacteroides* and *Prevotella* are negatively correlated with fat mass development in diet-induced obese mice^38^. Relevant to our study, within *Bacteroides* genus, the observed enrichment of *B. acidifaciens* is associated with lower body weight, fat mass and improvement of insulin resistance in hepatic autophagy-related gene 7 (*Atg7*)-deficient mice^39^. Of note, *B. acidifaciens* promoted the expression of PPAR-α in epididymal adipose tissue^39^.

On the other hand, a high fat/high refined carbohydrates diet leads to a prevalence of Firmicutes^40^. In our study, the reduction of *Lactobacillus* genus following OEA treatment involved *L. murinus*, *L. johnsonii*, and in particular *L. gasseri* and *L. reuteri*. In humans and animals, weight modifications, either weight loss or gain, were significantly associated with different *Lactobacillus* species and strains, and these modifications are believed to be host-specific as reported by two meta-analyses^41,42^. Some studies have shown that in pigs, turkeys and rats, *L. reuteri* induced a significant weight gain^41,43,44^, and in some cases, even if there is no clear evidence, an association with obesity^45–47^. Thus, our findings suggest, in the opposite direction, an association between OEA-induced diminished weight gain and reduced abundance of some commensal bacteria, such as *Lactobacillus*, but not of *Bacteroides*, in particular *B. acidifaciens*. Overall, our results suggest that OEA sub-chronic treatment shifts gut microbiota towards a “lean-like phenotype”. Prediction analysis (PICRUSt) of the metabolic pathways by metagenomic data as well suggests that OEA-induced shift of microbial profiles produces significant modifications of microbiome functions, suggesting relevant consequences on host metabolism and intestinal immune homeostasis.

OEA treatment, by activating PPAR-α, not only induces food-satiety and a modest weight loss^19,34^, as confirmed by our results, but it also enhances lipid utilization by stimulating fatty acid uptake, intracellular transport and lipolysis, fat oxidation, and can also modulate lipid levels in tissues and circulation^17,34^. In addition, OEA participates in the physiologic inhibition of intestinal motility^48^ presumably in a PPAR-α independent manner^49^, enhances long-chain fatty acid uptake in enterocytes^39^, and increases intestinal epithelial cells resistance^50^. These metabolic and homeostatic effects of OEA may change the intestinal environment and the ecological fitness of bacterial community, favouring the enrichment of *Bacteroides* at the expenses of other genera, such as *Lactobacillus*. We cannot exclude though, that the rapid effect on body weight induced by OEA contributes to the microbiota modification. Further studies should be performed to understand the role of direct and/or indirect mechanisms on the intestinal microbiota.

Several lines of evidence suggest that endogenous ligands of PPAR-α exert a tonic inhibitory control over the induction of inflammatory responses^25^. Whereas the anti-inflammatory effects of the PPAR-α agonist palmitoylethanolamide (PEA) are well documented^20^, less studied is the direct contribution of OEA to the production of inflammatory or regulatory cytokines, even more so in the gut-associated lymphoid tissues.

We found that OEA profoundly affects the polarization of T_H_ lymphocytes in the Peyer’s patches towards an anti-inflammatory profile, in agreement with previous reports that indicated how stimulation of PPAR-α competes with other transcription factors that are major mediators of inflammatory response in lymphocytes, monocyte and macrophages^51,52^. Furthermore, it is well established that PPAR-α genetic deletion results in increased T_H_1/T_H_17 response of cells isolated from the intestine lamina propria at steady state conditions and the condition is exacerbated in colitis^53^. Our observation of the concomitant change in microbiota and Peyer’s patches environment *in vivo* suggests a double, if not related, effect of OEA in the intestine, that may be exploited to counteract obesity induced regional and systemic inflammation^54^, balancing the reduction of a tolerant milieu associated with skewing toward T_H_1/T_H_17 immune profile in the liver, adipose and muscle tissue^13^. Immune responses are orchestrated by the production of pro- and anti-inflammatory cytokines, as the lymphonodes in the Peyer’s patches must continuously distinguish between commensal bacteria and pathogens, with consequences on systemic immunity. Therefore, the fact that OEA is acting on Peyer’s patches immune cells decreasing pro-inflammatory cytokines production and, at the molecular level, transcription factors responsible for T_H_1 and T_H_17 differentiation may be exploited in order to modulate gut inflammation. OEA and PEA *in vivo* decrease plasma and cortical TNFα mRNA levels^55^ and our experiments add further knowledge to this observation: in our hands TNFα, that in turn induces CXCL1 and CXCL2 production, is down modulated when Peyer’s patches derived cells are stimulated with PHA, but not with LPS, suggesting that OEA is promoting a less inflammatory mileu, without significantly affecting innate immune response and the ability of neutrophils recall, when necessary. In the future, it would be of interest to check if OEA anti-inflammatory effect is present in all intestinal regions and associated to immune cells, as in fact these may not behave in the same way as cells isolated from Peyer’s patches^56^.

Several intriguing questions remain unanswered as for instance whether the homeostatic effects of OEA on Peyer’s patches immune cells responses and microbiota are independent phenomena or whether they influence each other. Our results can be explained by two equally valid hypotheses: (i) the induction of a tolerogenic milieu in itself determines differences in the microbiome, or (ii) OEA-induced changes in the microbiome primes anti-inflammatory responses. There is evidence that bioactive lipids (e.g. palmytoylethanolamide, anandamide, short chain fatty acids) participate in physiological processes correlated with the maintenance of gut-barrier function, inflammation and energy metabolism^20,57,58^. Dysregulation of the endocannabinoid system might play a crucial role in the etiopathogenesis of intestinal disorders^59^. Enhancing endogenous OEA synthesis could represent a new therapeutic strategy to treat such diseases.

Overall, the present study stimulates future investigation addressed at solving the causal effects, also measuring the microbial metabolites produced by OEA treatment, to provide further useful insights on role of OEA in the gut-brain axis.

## Methods

### Animals

SV129 male mice aged 8–12 weeks were bred in our colony in the animal facility where they were single-housed in macrolon cages at 20–24 °C. Mice were allowed free access to chow pellets (Mucedola, Milano Italy) and water, and kept on a 12-h-light/-dark cycle (light started at 7:00 am). All experimental manipulations were performed in strict compliance with the EEC recommendations for the care and use of laboratory animals (2010/63/EU) approved by the Animal Care Committee of the Dipartimento di Scienze della Salute, Universitá di Firenze (I). Ethical policy of the Universitá di Firenze complies with the Guide for the Care and Use of Laboratory Animals of the Council Directive of the European Community (2010/63/EU) and the Italian Decreto Legislativo 26 (13/03/2014). Every effort was made to minimize animal suffering and to reduce the number of animals used. Mice were handled for at least 4 days before experiments begun, to let them acclimatise to human contact. Body weight and food consumption were measured daily, starting the day before administration of OEA or vehicle. OEA (Tocris Bioscience, UK) was dissolved in saline/polyethylene glycol/Tween80 (90/5/5, v/v) and administered at a dose of 10 mg/kg i.p. 30 min before lights off, once daily for 11 days. The dose of OEA was within the range of i.p. doses that have been reported in the literature and that do not cause harmful or unwanted side effects^19,60–62^. Mice were weighted and food consumption measured in the morning, and faecal pellets collected right after.

### Faecal samples collection and bacterial genomic DNA extraction

Faeces was collected (i) at steady state (T0) from each mice group, (ii) after 11 days of OEA treatment (OEA_T11), and (iii) after 11 days of vehicle-treatment (Vehicle_T11). For each group of mice, the pellets were collected in sterile conditions and stored at −80 °C until extraction of nucleic acids. The bacterial genomic DNA extraction was carried out with DNeasy PowerLyzer PowerSoil Kit (Qiagen, Hilden, Germania) following the manufacturer’s instructions. DNA quality was assessed by gel electrophoresis and spectrophotometry, measuring OD 260/280.

### 16S Ribosomal RNA Gene Amplicons preparation and Illumina MiSeq sequencing

Library of 16S rRNA gene amplicons was prepared by IGA Technology Services (Udine, Italy) through amplification of the V3-V4 hypervariable region by using specific-barcoded primers with overhang adapters. The standard protocol was followed according to the 16S metagenomic sequencing library preparation guide from Illumina (Part #15044223 Rev. B; https://support.illumina.com/). Pooled V3-V4 amplicon libraries were sequenced using the Illumina MiSeq platform.

### Data Analysis

Sequence data are available at http://www.ebi.ac.uk/ena/data/view/ PRJEB26276, under the accession number PRJEB26276. The 300-bp paired-end reads obtained from Illumina MiSeq platform for each sample were demultiplexed and quality checked using FastQC 0.11.5. Reads were further processed using the MICCA pipeline (version 1.6, ttp://compmetagen.github.io/micca/)^63^. Briefly, Illumina paired-end reads was merged by using the mergepairs function. Forward and reverse primers was trimmed, and reads did not contain the forward or the reverse primer was discarded, as well as both sequences preceding the forward or succeeding the reverse primers was removed. Quality filtering were performed by using mica-filter (according to the maximum allowed expected error rate % reported in filterstats), truncating reads shorter than 300 nt (quality threshold = 18), in order to produce high-quality reads. Denovo sequence clustering, chimera filtering and taxonomy assignment were performed by micca-otu-denovo. Operational Taxonomic Units (OTUs) were assigned by clustering the sequences with a threshold of 97% pair-wise identity. The representative sequences were classified using the RDP classifier version 2.7 against RDP 11 database (update 5) of 16S rRNA. Template-guided multiple sequence alignment was performed using PyNAST (version 0.1) against the multiple alignment of the Greengenes 16S rRNA gene database (release 13_05) and filtering at 97% similarity. A total of 3570 Operational Taxonomic Units (OTUs) were assigned by clustering the sequences with a threshold of 97% pair-wise identity. OUT tables for each taxonomic level were created by mica-tabletotax and used for further statistical analysis.

For *Bacteroides* and *Lactobacillus* genera, depth at species level was obtained by sequence alignment using Basic Local Alignment Search Tool nucleotide (BLASTn) software in the National Center for Biotechnology Information (NCBI) database. The highest percentage of identity (Query cover 100–99% and Identity 99 or 95%). Expectation value (E-value) was used to select significant BLAST hits, keeping only outcomes with the lowest E-value (minimal E-value of 10^−3^).

Alpha diversity was estimated by observed OTUs (a measure of microbial richness), Shannon entropy (a measure of entropy accounting for both abundance and evenness of bacterial species), Dominance (1-Simpson index), and Equitability (Shannon diversity divided by the logarithm of number of taxa), by using Paleontological Statistics Software Package (PAST3 v.3.12). Beta diversity was performed to evaluate differences in overall bacterial communities by Principal Coordinates Analysis (PCoA) and Non-metric Multidimensional Scaling (NMDS), based on Bray-Curtis dissimilarities, using the phyloseq package of the R software suite. The significance of between-groups differentiation on Bray-Curtis dissimilarity was assessed by PERMANOVA using the adonis() function of the R package vegan with 999 permutations.

To infer the functional pathways (KEGG categories) that differ between mice groups (treated with OEA vs control mice), we applied Phylogenetic Investigation of Communities by Reconstruction of Unobserved States- PICRUSt^64^ on 16S rDNA sequencing data set. We obtained the final output from metagenome prediction as an annotated table of predicted gene family counts for each sample, where the encoded function of each gene family be orthologous groups or other identifiers such as KEGG orthologs (KOs).

Metagenomic biomarker discovery and related statistical significance were assessed by using the linear discriminant analysis (LDA) effect size (LEfSe) method, based on the bacterial relative abundances. In LEfSe, Kruskal–Wallis rank-sum test is used to identify significantly different taxa abundances among groups of mice, and LDA to estimate the size effect of each feature. An alpha significance level of 0.05, either for the factorial Kruskal-Wallis test among classes or for the pairwise Wilcoxon test between subclasses, was used. A size-effect threshold of 2.0 on the logarithmic LDA score was applied for discriminative microbial biomarkers. LEfSe was also performed on PICRUSt data to discover bacterial functional biomarkers among groups of mice based on different treatments.

For PICRUSt analysis, statistical analysis and visualization of results were performed by using STAMP package (Statistical Analysis of Metagenomic and other Profiles; version Profiles^65^; version 2.1.3). Multiple comparison testing was performed using non-parametric Kruskal Wallis test. Two-group comparison was performed by White’s non-parametric t-test, using an alpha of 0.05, with Storey false discovery rate correction.

### Peyer’s patches isolation and cytokines determination

Mice were sacrificed by cerebral dislocation without anaesthesia. Peyer’s Patches were freshly isolated from the intestine and transferred in a 15 ml tube with PBS + 1% Pen/Strep (Lonza, Germany) to prevent drying. Peyer’s patches were digested with collagenase D (1 mg/ml) (Sigma-Aldrich, USA) in a 37 °C and 5% CO_2_ incubator for 5 minutes. All Peyer’s patches were transferred in a 70 µm cell strainer (Falcon, USA) placed over 50 ml tube and mechanically dissociated. During dissociation filters were washed with RPMI + 1% PEN/Strep. Peyer’s patches-single cells flowed through the filter and were suspended in 15 ml of RPMI, 10% FBS, 1%Pen/Strep, 0,01% beta-mercaptoethanol, 1% L-glutammin, 1% Na-Pir and 1% Hepes (complete medium). Cells were centrifuged at 1300 rpm at room temperature for 10 minutes, then the supernatant was discarded and pellets were suspended in complete medium. Peyer’s patches cells were counted with Thomas chamber using trypan blue dye exclusion. Cells were plated at the concentration of 1 × 10^5^ cells/100 $\mu$l (1 × 10^6^ cells/ml) in 96-well plate in the presence of PHA (5 ug/ml), LPS (1 ug/ml) (Sigma-Aldrich, USA) or vehicle for 48 h, in a 37 °C and 5% CO_2_ incubator. After incubation cell supernatants were collected for the determination of the following soluble factors: IFNγ, IL4, IL6, IL10, IL17, TNFα, CXCL1, CXCL2, by Luminex Technologies (Procartaplex, Life Technologies, USA).

### RNA extraction

Peyer’s patches cells were transferred in QIAzol (QIAGEN, Germany) lysis buffer, for RNA extraction. RT-PCR was carried out on 0.5–1 ng total RNA using Quantitec Reversion Transcription kit (QIAGEN, Germany) as first-strand primer. Real-time quantitative PCR was performed using TaqMan Universal PCR Master Mix, according to the manufacturer’s instructions (ThermoFisher Scientific, USA). The cDNA fragments corresponding to mouse Tbet, Rorc, GATA3, cMaf and Foxp3 were amplified using specific pairs of primers (Life Technologies, USA). All samples were run in triplicate on 96-well using optical PCR plates (Life Technologies, USA). Data were expressed as arbitrary units relative to expression of the gene encoding ubiquitin. The reference gene for RT-PCR, ubiquitin, was selected among others normally in use that are not involved in the process under examination, e.g. GADPH-R, 16S rRNA. To date no published indications are available for PP. Differences were revealed by unpaired Student’s t test, and significance was set at P < 0.05.

## References

[1] Chung H (2012). Gut immune maturation depends on colonization with a host-specific microbiota. Cell.

[2] Hooper LV, Littman DR, Macpherson AJ (2012). Interactions between the microbiota and the immune system. Science.

[3] Sáez de Guinoa Julia, Jimeno Rebeca, Gaya Mauro, Kipling David, Garzón María José, Dunn‐Walters Deborah, Ubeda Carles, Barral Patricia (2018). CD1d‐mediated lipid presentation by CD11c + cells regulates intestinal homeostasis. The EMBO Journal.

[4] Jung C, Hugot JP, Barreau F (2010). Peyer’s Patches: The Immune Sensors of the Intestine. International journal of inflammation.

[5] Kamada N, Seo SU, Chen GY, Nunez G (2013). Role of the gut microbiota in immunity and inflammatory disease. Nature reviews. Immunology.

[6] Bruchard M, Boidot R, Ghiringhelli F, Vegran F (2015). Transcriptome analysis of TH2 CD4(+) T cells differentiated from wild-type and NLRP3KO mice. Genomics data.

[7] Sampson Timothy R., Debelius Justine W., Thron Taren, Janssen Stefan, Shastri Gauri G., Ilhan Zehra Esra, Challis Collin, Schretter Catherine E., Rocha Sandra, Gradinaru Viviana, Chesselet Marie-Francoise, Keshavarzian Ali, Shannon Kathleen M., Krajmalnik-Brown Rosa, Wittung-Stafshede Pernilla, Knight Rob, Mazmanian Sarkis K. (2016). Gut Microbiota Regulate Motor Deficits and Neuroinflammation in a Model of Parkinson’s Disease. Cell.

[8] Strati F (2017). New evidences on the altered gut microbiota in autism spectrum disorders. Microbiome.

[9] Calvani R (2018). Of Microbes and Minds: A Narrative Review on the Second BrainAging. Frontiers in medicine.

[10] Marietta E, Horwath I, Taneja V (2018). Microbiome, Immunomodulation, and the Neuronal System. Neurotherapeutics: the journal of the American Society for Experimental NeuroTherapeutics.

[11] Riccio P, Rossano R (2018). Diet, Gut Microbiota, and Vitamins D+A in Multiple Sclerosis. Neurotherapeutics: the journal of the American Society for Experimental NeuroTherapeutics.

[12] Zhao L (2018). Gut bacteria selectively promoted by dietary fibers alleviate type 2 diabetes. Science.

[13] McPhee JB, Schertzer JD (2015). Immunometabolism of obesity and diabetes: microbiota link compartmentalized immunity in the gut to metabolic tissue inflammation. Clinical science.

[14] Kim KA, Gu W, Lee IA, Joh EH, Kim DH (2012). High fat diet-induced gut microbiota exacerbates inflammation and obesity in mice via the TLR4 signaling pathway. PloS one.

[15] Monk JM (2012). Dietary n-3 polyunsaturated fatty acids (PUFA) decrease obesity-associated Th17 cell-mediated inflammation during colitis. PloS one.

[16] Cani PD (2016). Endocannabinoids–at the crossroads between the gut microbiota and host metabolism. Nature reviews. Endocrinology.

[17] Piomelli D (2013). A fatty gut feeling. Trends in endocrinology and metabolism: TEM.

[18] Fu J, Kim J, Oveisi F, Astarita G, Piomelli D (2008). Targeted enhancement of oleoylethanolamide production in proximal small intestine induces across-meal satiety in rats. American journal of physiology. Regulatory, integrative and comparative physiology.

[19] Rodriguez de Fonseca F (2001). An anorexic lipid mediator regulated by feeding. Nature.

[20] Russo Roberto, Cristiano Claudia, Avagliano Carmen, De Caro Carmen, La Rana Giovanna, Raso Giuseppina, Canani Roberto, Meli Rosaria, Calignano Antonio (2017). Gut-brain axis: Role of lipids in the regulation of inflammation, pain and CNS diseases. Current Medicinal Chemistry.

[21] Artmann A (2008). Influence of dietary fatty acids on endocannabinoid and N-acylethanolamine levels in rat brain, liver and small intestine. Biochimica et biophysica acta.

[22] Hansen HS (2014). Role of anorectic N-acylethanolamines in intestinal physiology and satiety control with respect to dietary fat. Pharmacological research.

[23] Fu J (2007). Food intake regulates oleoylethanolamide formation and degradation in the proximal small intestine. The Journal of biological chemistry.

[24] Izzo AA (2010). Basal and fasting/refeeding-regulated tissue levels of endogenous PPAR-alpha ligands in Zucker rats. Obesity.

[25] Piomelli D, Sasso O (2014). Peripheral gating of pain signals by endogenous lipid mediators. Nature neuroscience.

[26] Ribeiro A (2015). A Potent Systemically Active N-Acylethanolamine Acid Amidase Inhibitor that Suppresses Inflammation and Human Macrophage Activation. ACS chemical biology.

[27] Schwartz GJ (2008). The lipid messenger OEA links dietary fat intake to satiety. Cell metabolism.

[28] Hug Hubert, Mohajeri M., La Fata Giorgio (2018). Toll-Like Receptors: Regulators of the Immune Response in the Human Gut. Nutrients.

[29] Wang Y (2010). Lymphotoxin beta receptor signaling in intestinal epithelial cells orchestrates innate immune responses against mucosal bacterial infection. Immunity.

[30] Oestreich KJ, Weinmann AS (2012). Master regulators or lineage-specifying? Changing views on CD4+ T cell transcription factors. Nature reviews. Immunology.

[31] Yao Y (2015). Tr1 Cells, but Not Foxp3+ Regulatory T Cells, Suppress NLRP3 Inflammasome Activation via an IL-10-Dependent Mechanism. Journal of immunology.

[32] Barros MH, Hauck F, Dreyer JH, Kempkes B, Niedobitek G (2013). Macrophage polarisation: an immunohistochemical approach for identifying M1 and M2 macrophages. PloS one.

[33] Suarez J (2014). Oleoylethanolamide enhances beta-adrenergic-mediated thermogenesis and white-to-brown adipocyte phenotype in epididymal white adipose tissue in rat. Disease models & mechanisms.

[34] Guzman M (2004). Oleoylethanolamide stimulates lipolysis by activating the nuclear receptor peroxisome proliferator-activated receptor alpha (PPAR-alpha). The Journal of biological chemistry.

[35] Ley RE (2005). Obesity alters gut microbial ecology. Proceedings of the National Academy of Sciences of the United States of America.

[36] Ley RE, Turnbaugh PJ, Klein S, Gordon JI (2006). Microbial ecology: human gut microbes associated with obesity. Nature.

[37] Terrapon N, Lombard V, Gilbert HJ, Henrissat B (2015). Automatic prediction of polysaccharide utilization loci in Bacteroidetes species. Bioinformatics.

[38] Neyrinck AM (2011). Prebiotic effects of wheat arabinoxylan related to the increase in *Bifidobacteria, Roseburia* and *Bacteroides/Prevotella* in diet-induced obese mice. PloS one.

[39] Yang JY (2017). Gut commensal *Bacteroides acidifaciens* prevents obesity and improves insulin sensitivity in mice. Mucosal immunology.

[40] Tilg H, Moschen AR, Kaser A (2009). Obesity and the microbiota. Gastroenterology.

[41] Million M (2012). Comparative meta-analysis of the effect of *Lactobacillus* species on weight gain in humans and animals. Microbial pathogenesis.

[42] Crovesy L, Ostrowski M, Ferreira D, Rosado EL, Soares-Mota M (2017). Effect of *Lactobacillus* on body weight and body fat in overweight subjects: a systematic review of randomized controlled clinical trials. International journal of obesity.

[43] Chang YH (2001). Selection of a potential probiotic *Lactobacillus* strain and subsequent *in vivo* studies. Antonie van Leeuwenhoek.

[44] Lu YC, Yin LT, Chang WT, Huang JS (2010). Effect of *Lactobacillus reuteri* GMNL-263 treatment on renal fibrosis in diabetic rats. Journal of bioscience and bioengineering.

[45] Armougom F, Henry M, Vialettes B, Raccah D, Raoult D (2009). Monitoring bacterial community of human gut microbiota reveals an increase in *Lactobacillus* in obese patients and Methanogens in anorexic patients. PloS one.

[46] Million M (2012). Obesity-associated gut microbiota is enriched in *Lactobacillus reuteri* and depleted in *Bifidobacterium animalis* and *Methanobrevibacter smithii*. International journal of obesity.

[47] Million M (2013). Correlation between body mass index and gut concentrations of *Lactobacillus reuteri, Bifidobacterium animalis, Methanobrevibacter smithii* and *Escherichia coli*. International journal of obesity.

[48] Capasso R (2005). Fatty acid amide hydrolase controls mouse intestinal motility *in vivo*. Gastroenterology.

[49] Cluny NL, Keenan CM, Lutz B, Piomelli D, Sharkey KA (2009). The identification of peroxisome proliferator-activated receptor alpha-independent effects of oleoylethanolamide on intestinal transit in mice. Neurogastroenterology and motility: the official journal of the European Gastrointestinal Motility Society.

[50] Karwad MA (2017). Oleoylethanolamine and palmitoylethanolamine modulate intestinal permeability *in vitro* via TRPV1 and PPARalpha. FASEB journal: official publication of the Federation of American Societies for Experimental Biology.

[51] Hines IN, Kremer M, Moore SM, Wheeler MD (2018). Impaired T cell-mediated hepatitis in peroxisome proliferator activated receptor alpha (PPARalpha)-deficient mice. Biological research.

[52] Yang L (2016). Oleoylethanolamide exerts anti-inflammatory effects on LPS-induced THP-1 cells by enhancing PPARalpha signaling and inhibiting the NF-kappaB and ERK1/2/AP-1/STAT3pathways. Scientific reports.

[53] Manoharan I (2016). Homeostatic PPARalpha Signaling Limits Inflammatory Responses to Commensal Microbiota in the Intestine. Journal of immunology.

[54] Mraz M, Haluzik M (2014). The role of adipose tissue immune cells in obesity and low-grade inflammation. The Journal of endocrinology.

[55] Sayd A., Anton M., Alen F., Caso J. R., Pavon J., Leza J. C., Rodriguez de Fonseca F., Garcia-Bueno B., Orio L. (2014). Systemic Administration of Oleoylethanolamide Protects from Neuroinflammation and Anhedonia Induced by LPS in Rats. International Journal of Neuropsychopharmacology.

[56] Magnuson Aaron M., Fouts Josephine K., Regan Daniel P., Booth Andrea D., Dow Steve W., Foster Michelle T. (2018). Adipose tissue extrinsic factor: Obesity-induced inflammation and the role of the visceral lymph node. Physiology & Behavior.

[57] Acharya N (2017). Endocannabinoid system acts as a regulator of immune homeostasis in the gut. Proceedings of the National Academy of Sciences of the United States of America.

[58] Sun M (2018). Microbiota-derived short-chain fatty acids promote Th1 cell IL-10 production to maintain intestinal homeostasis. Nature communications.

[59] Pesce M (2018). Endocannabinoid-related compounds in gastrointestinal diseases. Journal of cellular and molecular medicine.

[60] Costa A (2018). Histamine-deficient mice do not respond to the antidepressant-like effects of oleoylethanolamide. Neuropharmacology.

[61] Yang LC (2015). Chronic oleoylethanolamide treatment improves spatial cognitive deficits through enhancing hippocampal neurogenesis after transient focal cerebral ischemia. Biochemical pharmacology.

[62] Provensi G (2014). Satiety factor oleoylethanolamide recruits the brain histaminergic system to inhibit food intake. Proceedings of the National Academy of Sciences of the United States of America.

[63] Albanese D, Fontana P, De Filippo C, Cavalieri D, Donati C (2015). MICCA: a complete and accurate software for taxonomic profiling of metagenomic data. Scientific reports.

[64] Langille MG (2013). Predictive functional profiling of microbial communities using 16S rRNA marker gene sequences. Nature biotechnology.

[65] Parks DH, Tyson GW, Hugenholtz P, Beiko RG (2014). STAMP: statistical analysis of taxonomic and functional profiles. Bioinformatics.

